# Phenotypic- and Genotypic-Resistance Detection for Adaptive Resistance Management in *Tetranychus urticae* Koch

**DOI:** 10.1371/journal.pone.0139934

**Published:** 2015-11-06

**Authors:** Deok Ho Kwon, Taek-Jun Kang, Young Ho Kim, Si Hyeock Lee

**Affiliations:** 1 Research Institute of Agriculture and Life Sciences, Seoul National University, Seoul 151–921, Republic of Korea; 2 Department of Horticultural Crop Research, National Institute of Horticultural and Herbal Science, RDA, Jeonju 565–852, Republic of Korea; 3 Department of Agricultural Biotechnology, Seoul National University, Seoul 151–921, Republic of Korea; University of Crete, GREECE

## Abstract

Rapid resistance detection is necessary for the adaptive management of acaricide-resistant populations of *Tetranychus urticae*. Detection of phenotypic and genotypic resistance was conducted by employing residual contact vial bioassay (RCV) and quantitative sequencing (QS) methods, respectively. RCV was useful for detecting the acaricide resistance levels of *T*. *urticae*, particularly for on-site resistance detection; however, it was only applicable for rapid-acting acaricides (12 out of 19 tested acaricides). QS was effective for determining the frequencies of resistance alleles on a population basis, which corresponded to 12 nonsynonymous point mutations associated with target-site resistance to five types of acaricides [organophosphates (monocrotophos, pirimiphos-methyl, dimethoate and chlorpyrifos), pyrethroids (fenpropathrin and bifenthrin), abamectin, bifenazate and etoxazole]. Most field-collected mites exhibited high levels of multiple resistance, as determined by RCV and QS data, suggesting the seriousness of their current acaricide resistance status in rose cultivation areas in Korea. The correlation analyses revealed moderate to high levels of positive relationships between the resistance allele frequencies and the actual resistance levels in only five of the acaricides evaluated, which limits the general application of allele frequency as a direct indicator for estimating actual resistance levels. Nevertheless, the resistance allele frequency data alone allowed for the evaluation of the genetic resistance potential and background of test mite populations. The combined use of RCV and QS provides basic information on resistance levels, which is essential for choosing appropriate acaricides for the management of resistant *T*. *urticae*.

## Introduction

Over 100 acaricides, classified into 14 groups based on their modes of action, have been developed and registered for commercial use in the control of two-spotted spider mites, *Tetranychus urticae*, on horticulture and agricultural crops worldwide [1]. However, the *T*. *urticae* population has developed resistance to nearly all acaricides due to the mite’s short life cycle, high biotic potential and arrhenotokous reproduction [2].

In Korea, approximately 52 acaricides have been commercialized and intensively used to control *T*. *urticae* on various crops for 50 years, resulting in the rapid development of acaricide resistance in field populations [3]. Resistance management has mainly relied on the use of new acaricides with novel modes of action to control resistant populations. With the introduction of new acaricides, resistant mite populations were temporarily controlled. Soon after their continuous use, however, mites developed additive resistance, resulting in populations having multiple resistance mechanisms to different types of acaricides. Thus, a more systematic resistance management, based on the discrete selection and appropriate use of new acaricides, is urgently required to alleviate the serious resistance problems in *T*. *urticae*.

Generally, two strategies have been suggested for resistance management: preventative and adaptive management. Preventative resistance management includes several tactics, such as rotation, refuge and high-dose applications based on theoretical predictions [4], but these tactics are not employable in populations with high levels of multiple resistance. In the adaptive resistance management strategy, management actions are decided based on detailed information on the actual frequencies of resistance alleles [4]. In addition, this strategy requires additional information on the heredity and fitness of resistance alleles [5]. Adaptive management enables the estimation of resistance levels for each pesticide and this is used to determine the most practical plan of count measure for resistant pest management. However, adaptive resistance management can only be realized after the establishment of sensitive monitoring and diagnostic tools to determine resistance levels or resistance allele frequencies.

Various resistance monitoring methods, such as the slide dip method [6], leaf dip method [6], whole-plant-residual bioassay [7], a practitioner-assessable bioassay [8], spray method [9, 10], and residual contact vial bioassay (RCV) [11], have been designed and are widely used to determine resistance levels. Among these, RCV was developed as a less technique-dependent bioassay that can be conveniently used by practitioners or farmers in the field for monitoring acaricide resistance [12]. In the initial report by Kwon et al. [11], it was demonstrated that RCV could discriminate the presence of resistance to abamectin and tebufenpyrad in field populations in 8 h. However, because the pre-determination of the diagnostic dose of each acaricide is a prerequisite for the use of RCV, extended estimations of diagnostic doses for major acaricides currently used in the field are essential for the wider application of RCV in resistance monitoring.

As an alternative to the evaluation of phenotypic resistance levels based on traditional bioassays, various molecular markers for resistance detection have been identified through extensive resistance mechanism studies. The recent completion of *T*. *urticae* genome analysis has greatly accelerated the identification of several genetic markers conferring resistance to new acaricides [13, 14]. At present, several qualitative markers (nonsynonymous mutations) resulting in amino acid changes in the acaricide target proteins have been identified in the acetylcholinesterase (*Tuace*), voltage sensitive sodium channel (*Tuvssc*), glutamate-gated chloride channel (*TuGluCl*), chitin synthase 1 (*TuCHS*) and cytochrome b (*TuCytB*) genes (Table 1) [15, 16]. Five point mutations (G228S, A309S, A391T, G436A and F439W) have also been identified in the catalytic triad and peripheral anionic sites of *Tuace* [9, 17–19]. Three point mutations (1022V, A1366D and F1538) associated with pyrethroid resistance were found on *Tuvssc* [20, 21]. Two point mutations (G323D and G326E) in two different types of glutamate-gated chloride channels (GluCl1 and GluCl3, respectively) were identified to confer abamectin resistance [22, 23]. A point mutation (I1017F) associated with etoxazole resistance was identified in *TuCHS* [24]. Five point mutations (G126S, I136T, S141F, D161G and P262T) associated with bifenazate resistance were found in *TuCytB* [25, 26]. The role of each mutation in resistance has been suggested either by toxicity analysis or by crossing with susceptible strains.

**Table 1:** Transition and transversion of point mutation associated acaricides resistance in *Tetranychus urticae*.

Acaricides	Target protein	Accession number	Amino acid position	Wild type	Mutant	Abbreviation	Substituted nucleotide	Pattern of substitutions
Organophosphate	AChE	tetur19g00850	228	Gly(**G**GC)	S(**A**GC)	G228S	G→A	Transition
				Gly(**G**GC)	A(**T**GC)	G228A	G→T	Transversion
			309	Ala(**G**CT)	Ser(**T**CT)	A309S	G→T	Transversion
			391	Ala(**G**CA)	Thr(**A**CA)	A391T	G→A	Transition
			436	Gly(G**G**A)	Ala(G**C**A)	G436A	G→C	Transversion
			439	Phe(T**TT**)	Trp(T**GG**)	F439W	T→G	Transversion
				Phe(T**T**T)	Tyr(T**A**T)	F439Y	T→A	Transversion
Pyrethroid	VSSC	Tetur34g00970	1022	Leu(**C**TC)	Val(**G**TC)	L1022V	C→G	Transversion
			1376	Ala(G**C**T)	Asp(G**A**T)	A1376D	C→A	Transversion
			1704	Phe(**T**TC)	Ile(**A**TC)	F1704I	T→A	Transversion
Abamectin	GluCl1	Tetur02g04080	323	Gly(G**G**T)	Asp(G**A**T)	G323D	G→A	Transition
	GluCl3	Tetur10g03090	326	Gly(G**G**A)	Glu(G**A**A)	G326E	G→A	Transition
Etoxazole	CHS	Tetur03g08510	1017	Ile(**A**TT)	Phe(**T**TT)	I1017F	A→T	Transversion
Bifenazate	CytB	YP_001795379.1	126	Gly(**G**GA)	Ser(**A**GA)	G126S	G→A	Transition
			136	Ile(A**T**T)	Thr(A**C**T)	I136T	T→C	Transition
			141	Ser(T**C**T)	Phe(T**T**T)	S141F	C→T	Transition
			161	Asp(GAT)	Gly(GGT)	D161G	A→G	Transition
			262	Pro(**C**CT)	Thr(**A**CT)	P262T	C→A	Transversion

Various PCR-based molecular detection techniques, including PASA (PCR Amplification of Specific Alleles) [27], bi-PASA [28], rt-PASA [29], real time PCR with allele specific TaqMan Probes [30], and Quantitative Sequencing (QS) [31] have been developed for the simple, rapid and accurate allele frequency estimation of point mutations conferring pesticide resistance in individual and population samples of pesticide-resistant mites and insects [32–36]. Among these, the quantitative sequencing (QS) method was determined to be efficient and cost-effective in simultaneously estimating the resistance allele frequencies in a large number of pooled genomic DNA samples of *T*. *urticae* [25, 37].

In this study, using basic resistance diagnostic tools for the implementation of an adaptive management system, we tested the performances of the RCV and QS methods in determining resistance levels and corresponding resistance allele frequencies, respectively, in 12 strains of *T*. *urticae*. The diagnostic doses for 12 acaricides were estimated for RCV. In addition, equations for the frequency prediction of 12 point mutations associated with resistance to five different acaricide groups were established for QS. To determine how the bioassay data obtained by RCV and the resistance allele frequency data obtained by QS are related, thereby evaluating the applicability of each mutation allele as a resistance marker, we conducted correlation analyses for the data obtained from 12 strains of *T*. *urticae*.

## Materials and Methods

### Acaricides

All acaricides were technical grade and were obtained from the following sources: fenothiocarb (99.8%, Sigma Aldrich, Saint Louis, Mo) monocrotophos (99.5%, Chem Service, West Chester, PA), omethoate (97.0%, Sigma Aldrich), endosulfan (99.4%, Sigma Aldrich), bifenthrin (99.0%, Sigma Aldrich), fenpropathrin (99.5%, Chem Service, West Chester, PA), Abamectin (97.6%, Sigma Aldrich), etoxazole (98.5%, Sigma Aldrich), fenbutatin oxide (97.7%, Sigma Aldrich), chlorfenapyr (99.6%, Sigma Aldrich), flufenoxuron (98.4%, Sigma Aldrich), amitraz (96.8%, Sigma Aldrich), pyridaben (99.7%, Sigma Aldrich), tebufenpyrad (99.8%, Sigma Aldrich), spiromesifen (99.8%, Sigma Aldrich), (EZ)-cyenopyrafen (97.3%, Sigma Aldrich), cyflumetofen (96.0%, Wako Pure Chemical Industries, Ltd., Japan), bifenazate (99.9%, Sigma Aldrich) and dicofol (97.2%, Sigma Aldrich).

### Mite strains and rearing conditions

A total of 12 strains were used in this study. Six strains (UD, PyriF, AD, FenR and AbaR) were maintained in an insectary for over eight years. The remaining six strains were initially collected as one of regular survey for pest monitoring on rose cultivation area supported by Rural Developmental Administration after getting owner’s permission in Korea in 2013 (Table 2). All mites were reared on kidney bean plants (2-wk old, *Phaseolus vulgaris* variety *humilis*). All mite strains were maintained at 55±5% relative humidity (RH), a photoperiod of 16:8 (L:D) h and 25±1°C, except for the AbaR strain that was reared at 28±1°C.

**Table 2:** *Tetranychus urticae* collection sites.

No.	Strains	Collection sites	Body color	Host plants	Coordinates	Date	Remarks
**1**	UD	Ulleung-eup, Ulleung-gun, Gyeongbuk	Green	Greater celandine		Jun., 2006	Susceptible
**2**	PyriF	-	Green	-		^a^* Jul., 2005	Susceptible
**3**	AD	Andong-si, Gyeongbuk	Green	Apple tree		Aug., 2006	OP resistance
**4**	FenR	Yeongcheon-si, Gyeongbuk	Green	Apple tree		Aug., 2006	Pyrethroid resistance
**5**	PTF	Jinwi-myeon, Peongtaek-si, Gyeonggi	Green	Rose		Oct., 2007	Abamectin resistance
**6**	13GG_GY_G1	Deokyang-gu, Goyang-si, Gyeonggi	Green	Rose	37°40'32.20"N 126°51'33.71"E	Jun. 26, 2013	Field collected
**7**	13GG_SW_G1	Gweonsun-gu, Suweon-si, Gyeonggi	Green	Rose	37°15'09.84"N 126°58'32.22"E	Aug. 12, 2013	Field collected
**8**	13JB_GJ_G1	Keumgu-myeon, Gimje, Jeonbuk	Green	Rose	35°48'87.92"N 126°49'03.06"E	Oct. 29, 2013	Field collected
**9**	AbaR	Jangan-gu, Suweon-si, Gyeonggi	Red	Rose	37°15'09.84"N 126°58'32.22"E	Oct., 2007	Abamectin resistance
**10**	13GG_GY_R1	Deokyang-gu, Goyang-si, Gyeonggi	Red	Rose	37°40'32.20"N 126°51'33.71"E	Jun. 26, 2013	Field collected
**11**	13GG_PJ_R1	Jori-eup, Paju-si, Gyeonggi	Red	Rose	37°45'16.23"N 126°49'27.57"E	Jun. 26, 2013	Field collected
**12**	13CB_JC_R1	Yiweol-myeon, Jinchun-gun, Chungbuk	Red	Rose	36°55'23.99"N 127°25'49.91"E	Sep. 03, 2013	Field collected

^a^ Asterisk represents the date of acquisition from research institutions.

### Genomic DNA extraction

The genomic DNA was extracted from 50–100 females using the Qiagen DNeasy^®^ Blood & Tissue kit (Qiagen, GmBH, Germany) following the manufacturer’s instructions. Briefly, the mites were homogenized using pairs of disposable 1.5-ml polypropylene tubes and pestles (Bel-Art Scienceware, Wayne, NJ) with 100 μl lysis buffer. The homogenate was incubated with 100 μg of proteinase K at 56°C for 1 h for lysis. The lysate was transferred to DNA binding columns was centrifuged for 1 min and washed with 500 μl washing buffer. The genomic DNA in the DNA binding column was eluted with nuclease free water and stored at -20°C until use.

### Species identification and construction of the phylogenetic tree

A phylogenetic tree was constructed from mitochondrial cytochrome c oxidase I (mtCOI) gene sequences of 12 Korean strains and 51 representative nucleotide sequences of Tetranychus *spp*. which have been deposited in GenBank (S1 Table). The amplification of the mtCOI partial fragment (ca. 804-bp) and sequencing were conducted according to the methods of Kwon et al. [38]. A total of 63 partial mtCOI nucleotide sequences (598-bp) were aligned by CLUSTALW [39] and the empty block was completely removed using GBLOCK [40]. The best model was chosen as GTR+G+I using MEGA5.0 [41]. The fixed value of discrete Gamma distribution (+G) was 0.85 with 5 rate categories and the evolutionarily invariable (+I) was 0.48. The assumed values of transition/transversion bias were 2.05. The phylogenetic tree was constructed with Maximum likelihood methods using PhyML 3.0 with the approximate likelihood ratio test method [42].

### Determination of toxicity parameters and diagnostic doses for RCV and leaf dipping

The toxicity parameters [median lethal dose (LD_50_) and LD_90_] were determined by RCV for 19 acaricides, based on the methods of Kwon et al. [11]. Briefly, 100 μl aliquots of acaricide solutions were dissolved in acetone at various concentrations (0.3 ~ 1,000 ppm of stock solution converted to 1.8 × 10^−3^ ~ 6.1 μg^-1^cm^2^ in 5-ml glass vials) and used to coat the inside walls of 5-ml glass vials (Taeshin Bio Science, Seoul, Korea) using a rolling wave rotator (Eberbach, Ann Arbor, MI) for 1 h under a fume hood. Mites (15–18) were placed in each vial and mortality was determined at 8 h post-treatment with three replications. Any mites that were unable to move their bodies in 2–3 sec were considered dead. The LD_50_ and LD_90_ values were determined by probit analysis using IBM SPSS statistics ver. 20.0 (IBM Corp., NY). The diagnostic dose (D/D) was arbitrarily designated as the two-fold dose of the LD_90_.

For etoxazole, the toxicity parameters and diagnostic dose were determined using the leaf dipping method of Lee et al. [43] with a slight modification. Briefly, a soybean leaf disc (30-mm diameter) was placed on a water-soaked cotton pad and 40–70 females were allowed to oviposit eggs on the leaf disc for 5 h at 28°C. The working solution of etoxazole was prepared by dissolving technical grade etoxazole (98.5%, Sigma Aldrich) and further diluting with water containing 0.01% Triton X-100 (0.003 ~ 0.05 ppm final). For control treatment, the 0.01% Triton X-100 solution without etoxazole was used. After removing the females, the leaf disc with eggs was soaked in the etoxazole solution for 10 sec and dried under a fume hood for 1 h. The etoxazole-treated leaf was incubated at 24°C on the water-soaked cotton. The mortality was determined at 6~7 days post-treatment and any eggs not hatched were considered dead. The determination of the LC_50_, LC_90_ and diagnostic concentration (D/C) was based on the same method described above.

### Spray bioassay with recommended concentrations and comparison of their toxicity with those of RCV

For the spray bioassay, six laboratory strains (UD, PyriF, AD, FenR, PTF and AbaR) were independently treated with recommended concentrations of six commercial acaricides (monocrotophos—240 ppm, Dongbu Farm Hannong, Korea; fenpropathrin—50 ppm, Dongbangagro, Korea; abamectin—6.03 ppm, Syngenta, Korea; tebufenpyrad—25 ppm, Syngenta, Korea; cyflumetofen—100 ppm, Dongbangagro, Korea; and bifenazate—120 ppm, Syngenta, Korea). Each acaricide was diluted with water to the recommended concentration following manufacturer’s instruction. The spray was conducted according to the previous method with slight modifications [44]. The leaf disc (2.5-cm diameter) was placed on the top of water-soaked cotton pad and 9–22 female adults (17.5 females on average) were infested. Each acaricide solution was sprayed 30 times to the leaf disc with mites using a mini spray (Dongbang Plastic, Seoul, South Korea) apart from 40–50 cm. The residual acaricide solution was completely dried under a fume hood for 30–60 min. The mite that did not move the distance of its body size after prodding was considered as dead. Each experiment was conducted with three replications and mortality was determined at 24 h after treatment. The resulting mortalities from six mite strains were correlated with those obtained by RCV with the diagnostic doses.

### Establishment of QS protocols

Four QS linear regressions (G228S and F439W in *Tuace*, L1022V in *Tuvssc*, and G323D in *TuGluCl1*), to determine the organophosphate, pyrethroid and abamectin acaricide resistance allele frequencies, were established in previous studies [35, 45]. Additional QS regression equations were established to predict the frequencies of various mutations (A1376D and F1704I in *Tuvssc*; G326E in *TuGluCl3*; I1017F in *TuCHS1*; and G126S and P262T in *TuCytB*). Briefly, the partial genomic fragment (390 ~ 1200-bp) of a target gene fragment containing either the susceptible or resistant allele was amplified with 250 μM dNTPs, 5 μM primers (S2 Table), 5 μl gDNA template and 1 unit of GenAll Taq DNA polymerase (GeneAll Biotechnology Co., Ltd., Korea) in a 20 μl reaction following a thermal cycler program of 35 cycles of 94°C/30 s, 56~58°C/30 s, and 72°C/90 s. The PCR fragments were TA-cloned into pGEM-T easy vector^Ⓡ^ (Promega, Madison, WI), according to manufacturer’s protocol. The resulting plasmid DNA was used as the template for the PCR amplification of respective standard DNA templates for QS under the same conditions described above. The amount of amplified template DNA was quantified and normalized using the Low DNA mass ladder (Invitrogen) on agarose gel electrophoresis. Standard DNA templates with a series of different allele frequencies were generated by mixing each reference PCR product with its opposite allele in following molar ratios: 0:10, 1:9, 3:7, 5:5, 7:3, 9:1, and 0:10 (resistant allele: susceptible allele at each mutation site). All other procedures for the construction of the QS protocol were the same as those reported previously [35, 45]. The sequences of the resulting PCR products were analyzed by nested primers listed in S2 Table.

### Statistical analysis

The correlation analysis between the RCV mortality data vs. spray mortality data and the RCV mortality data vs. QS resistance allele frequency data were determined by Spearman correlation using IBM SPSS Statistics software (SPSS, Inc., Chicago, IL). A heat map and correlation matrix were drawn using R [46].

## Results

### Haplotype composition of the Korean population of *T*. *urticae*

**Fig 1:**
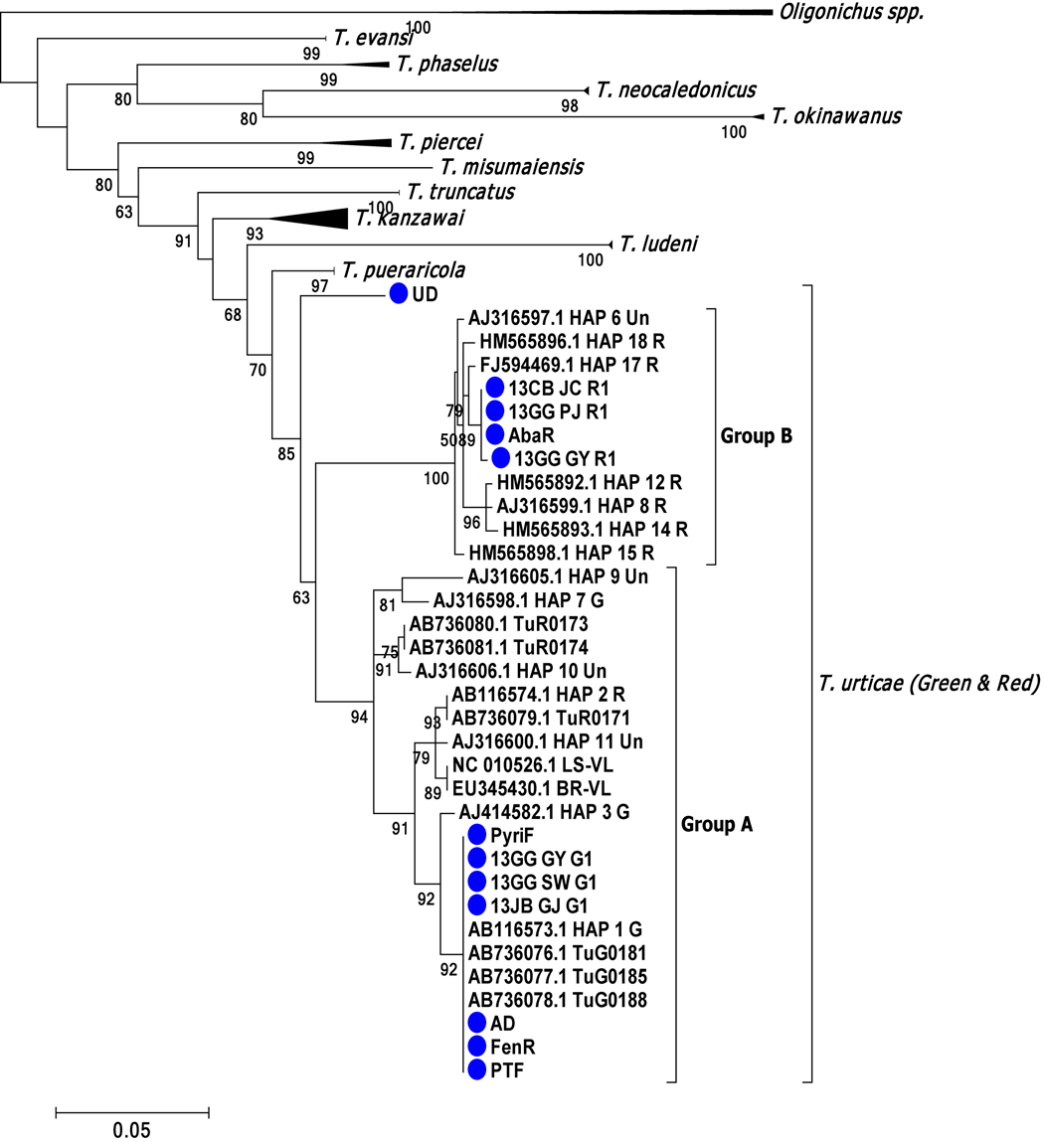
Phylogenetic tree of *Tetranychus* mites based on mtCOI partial sequences. Two *Oligonychus spp*. were used as outgroups. A maximum likelihood test was conducted and a bootstrap value over 50% is shown. The Korean populations are designated with blue circles.

The phylogenetic relationships of 12 Korean strains were analyzed along with other several Tetranychidae species (S1 Table). The tested *T*. *urticae* strains were largely divided into two groups (groups A and B, comprising red- and green-type mites) (Fig 1). Group A was commonly composed of both red- (AB736079.1 TuR0171, AB736080.1 TuR0173, AB736081.1 TuR0174 and AB116574.1 HAP 2R) and green-type mites, whereas Group B was only composed of red-type mites. Among the 12 Korean strains tested, seven strains (PyriF, AD, FenR, PTF, 13GG_GY_G1, 13GG_SW_G1 and 13JB_GJ_G1) were clustered within ‘haplotype 1’ (previously defined by [47]) as group A, known as the major green-type mites (Fig 1). Four strains (13CB_JC_R1, 13GG_PJ_R1, 13GG_GY_R1 and AbaR) were closely clustered with haplotype 17 (previously defined by [47]) as group B, known as red-type mites (Fig 1). As a result, both the green-type mites belonging to haplotype 1 and the red-type mites closely related with haplotype 17 were mainly distributed in the greenhouse rose cultivation areas in Korea.

### Determination of the diagnostic dose for RCV

The toxicity parameters for 18 acaricides belonging to 14 representative groups were determined using the RCV method with PyriF as a reference acaricide-susceptible strain. Although the UD strain exhibited higher susceptibility to all tested acaricides, it was not used as a reference susceptible strain because of its distant genetic background as evidenced from the phylogenetic analysis (Fig 1) [38]. The LD_50_ values of the PyriF strain for the 18 acaricides ranged from 2 × 10^−3^ to over 6.1 μg^-1^cm^2^ at 8 h post-treatment (Table 3). Both abamectin and chlorfenapyr showed the highest efficacy according to their LD_50_ values (approximately 2 × 10^−3^ μg^-1^cm^2^). In contrast, the chitin synthase inhibitors, etoxazole and flufenoxuron, showed the lowest efficacy with LD_50_ values over 6.1 μg^-1^cm^2^. In general, the acaricides with neurotoxic modes of action showed a rapid contact toxicity even at relatively low doses, while acaricides with growth regulator activity (i.e., as chitin synthase inhibitors working on immature stages) or metabolic toxicity revealed a relatively slow toxicity (Table 3).

**Table 3:** Toxicity parameter by residual contact vial bioassay to PyriF strains in *Tetranychus urticae*.

Group^a^	Acaricides	Target	N	Slope ± SE	*χ* ^*2*^	df	Toxicity levels (μg^-1^cm^2^)		
							LD_50_	LD_90_	D/D
1A1	Fenothiocarb	Nerve system	235	1.7 ± 0.2	16.2	(13)	0.252 (0.192–0.334)	1.41 (0.939–2.492)	2.8
1B	Monocrotophos	Nerve system	182	2.1 ± 0.3	25.5	(10)	0.055 (0.034–0.097)	0.22 (0.118–0.894)	0.441
1B	Omethoate	Nerve system	141	1.1 ± 0.3	6.4	(7)	0.008 (0.005–0.014)	0.11 (0.04–1.575)	0.219
2A	Endosulfan	Nerve system	232	1.4 ± 0.2	23.8	(13)	0.162 (0.097–0.261)	1.32 (0.697–4.056)	2.6
3A	Bifenthrin	Nerve system	286	1.7 ± 0.2	18.5	(16)	0.027 (0.021–0.035)	0.16 (0.107–0.255)	0.31
6	Abamectin	Nerve system	189	1.6 ± 0.2	20.1	(10)	0.002 (0.001–0.003)	0.01 (0.006–0.058)	0.023
10B	Etoxazole	Metabolic pathway	204	0.9 ± 0.4	5.4	(10)	246.3 (24—ND)	7912 (150.2—ND)	15824
10B	Etoxazole^b^	Metabolic pathway	625	3.3 ± 0.3	5.3	(3)	0.0083 (0.007–0.011)	0.021 (0.015–0.033)	0.041
12B	Fenbutatin oxide	Metabolic pathway	204	2.4 ± 0.3	9.4	(10)	2.0 (1.6–2.6)	7.03 (5.061–11.494)	14.1
13	Chlofenapyr	Metabolic pathway	228	2.6 ± 0.3	18.4	(13)	0.002 (0.0016–0.003)	0.01 (0.005–0.013)	0.014
15	Flufenoxuron	Metabolic pathway	204	ND^a^ ± ND^a^	ND^a^	(ND)	> 6.1 (ND—ND)	> 6.1 (ND—ND)	> 12.2
19	Amitraz	Nerve system	199	2.0 ± 0.2	6.3	(10)	0.39 (0.303–0.501)	1.7 (1.2–2.9)	3.4
21A	Pyridaben	Metabolic pathway	165	0.9 ± 0.1	8.7	(11)	1 (0.6–1.6)	28.7 (12.8–114.6)	57.4
21A	Tebufenpyrad	Metabolic pathway	287	1.4 ± 0.1	8.6	(16)	0.054 (0.039–0.073)	0.47 (0.303–0.846)	0.933
23	Spiromesifen	Metabolic pathway	204	1.8 ± 0.2	5.3	(10)	1.5 (1.2–2)	7.93 (5.2–15.0)	15.9
25	Cyenopyrafen	Metabolic pathway	193	2.1 ± 0.3	12.9	(10)	0.025 (0.019–0.032)	0.1 (0.07–0.161)	0.197
25	Cyflumetofen	Metabolic pathway	244	2.9 ± 0.3	12.3	(12)	0.112 (0.094–0.133)	0.31 (0.245–0.428)	0.618
UN	Bifenazate	Unknown	238	2.9 ± 0.3	2.3	(13)	0.083 (0.07–0.098)	0.23 (0.185–0.321)	0.464
UN	Dicofol	Unknown	185	1.0 ± 0.2	11.3	(10)	0.9 (0.6–1.8)	16.6 (5.7–156.7)	33.3

^a^ The grouping was based on the mode of action classification by Insecticide Resistance Action Committee (IRAC) (http://www.irac-online.org/documents/moa-structures-poster-english/?ext=pdf)

^b^ ND represents ‘not determined’

^c^ Determined by leaf dipping methods based on emergence rate. The unit of toxicity levels were ppm.

The LC_98 or 99_ or LD_98 or 99_ value has been suggested as a diagnostic dose that can kill all or nearly all susceptible individuals but few or no resistant individuals [48, 49]. However, as it is sometimes estimated by extrapolation of log-dose probit lines, such a value is prone to prediction error [48]. With this in mind, the diagnostic dose was arbitrarily set as a value two-fold greater than the LD_90_, which exhibited a significantly narrower range of the 95% confidence interval than LD_99_ (data not shown). The diagnostic doses for the 18 tested acaricides ranged from 0.014 to 15,824 μg^-1^cm^2^. Among these, the 11 acaricides that showed rapid contact toxicity were chosen for evaluating their efficacy against the 12 mite populations (Table 3). In the case of etoxazole, the resulting diagnostic dose was too high (over 3.05 μg^-1^cm^2^, corresponding 500 ppm in 100 μl) to form a viscous film over the inner surface of the treated vial, which trapped the infested mites. Therefore, RCV could not be employed and the efficacy of etoxazole was determined using the leaf-dipping method.

### Evaluation of acaricide susceptibility using RCV and the selection of appropriate acaricides

**Fig 2:**
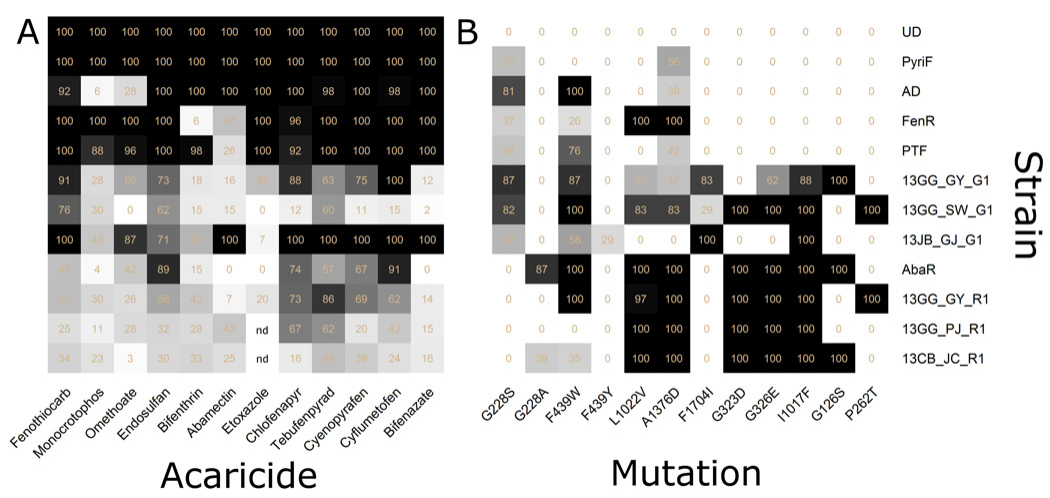
Heat maps of the mortalities and allele frequencies in 12 strains of *T*. *urticae*. (A) Heat map of the mortalities obtained by the diagnostic doses of RCV. (B) Heat map of the allele frequencies of 12 target site mutations determined by QS. The figure in each cell represents the actual value of color intensity and ‘nd’ in the empty cells represents a ‘not-determined value’. The actual values calculated by regression equation were listed in S6 Table.

Mortalities of 12 mite populations were determined using diagnostic doses of 12 acaricides. The UD and PyriF strains showed a 100% mortality to all tested acaricides at 8 h post-treatment (Fig 2A and S3 Table).

Using the diagnostic dose of fenothiocarb, seven strains (AD, FenR, PTF, 13GG_GY_G1, 13GG_SW_G1, 13JB_GJ_G1 and 13GG_GY_R1) exhibited mortality rates of 50 ~ 100%, whereas three strains (AbaR, 13GG_PJ_R1 and 13CB_JC_R1) showed 24.8 ~ 49.2% mortality rates. For monocrotophos, the FenR and PTF strains exhibited over 80% mortality, whereas the remaining 10 strains showed less than 40% mortality. Treatment with omethoate caused an 86.7 ~ 100% mortality rate in FenR, PTF and 13JB_GJ_G1 strains, whereas it resulted in a 0 ~ 56.3% mortality rate in the remaining seven strains. The monocrotophos-resistant AD strain exhibited the lowest mortality rates to organophosphate acaricides (5.9% and a 27.7% mortality rate to monocrotophos and omethoate, respectively) (Fig 2A and S3 Table). The diagnostic dose of endosulfan, an organochlorine acaricide, caused more than 60% mortality in seven mite populations but less than 60% mortality in three of the red-type mite populations. In the treatment with bifenthrin, a pyrethroid acaricide, all test populations, except for the AD and PTF strains, exhibited low mortality responses of less than 55%. The pyrethroid-resistant FenR strain showed the lowest mortality rate (6.3%) (Fig 2A and S3 Table). With the diagnostic dose of abamectin, only the AD and 13JB_GJ_G1 strains showed 100% mortality rate, whereas the remaining eight strains exhibited less than 50% mortality. The PTF and AbaR strains, known to be resistant to abamectin, exhibited mortality rates of 26.5% and 0%, respectively (Fig 2A and S3 Table). When treated with the diagnostic dose of etoxazole by the leaf dipping method, the AD, FenR and PyriF strains displayed 100% mortality, but the remaining five strains showed less than 50% mortality (Fig 2A and S3 Table). Chlorfenapyr treatment resulted in over 60% mortality in eight strains, whereas less than 15.6% mortality was observed in the remaining strains (13GG_SW_G1 and 13CB_JC_R1). Tebufenpyrad generally caused high mortalities (>60%) in eight strains, whereas the AbaR and 13CB_JC_R1 strains exhibited relatively lower mortality rates. Cyenopyrafen and cyflumetofen, belonging to group 25, caused over 60% mortality in seven strains, but less than 50% mortality in the 13GG_SW_G1, 13GG_PJ_R1 and 13CB_JC_R1 populations. Treatment of the diagnostic dose of bifenazate resulted in high mortalities in four strains but low mortalities (of less than 20%) in six strains.

In a summary, each mite population exhibited a varied mortality response when treated with diagnostic doses of the different acaricides by RCV, which indicates that the overall susceptibility profiles of a test mite population to different acaricides can be determined by RCV, once diagnostic doses are established. The susceptible reference strains (UD and PyriF) exhibited 100% mortalities, whereas the known acaricide-resistant strains (AD, FenR and PTF and AbaR) exhibited reduced mortality rates in response to the diagnostic doses of corresponding acaricides, confirming the potential of RCV in discriminating the reduced susceptibility levels in mite populations. In addition, a mite strain resistant to one acaricide also showed reduced mortalities to other acaricides, suggesting the possibility of cross-resistance and further implying that multiple resistance to different groups of acaricides is widespread in most mite populations. Diagnostic doses of six acaricides (fenothiocarb, endosulfan, chlorfenapyr, tebufenpyrad, cyenopyrafen and cyflumetofen) resulted in a 68.0~71.8% mortality rate (on average) in the test mite populations, whereas the remaining six acaricides (monocrotophos, omethoate, bifenthrin, abamectin, etoxazole and bifenazate) caused slightly reduced mortality rates (on average 36.5~59.3%).

### Validation of the reliability of resistance detection based on RCV

To verify the reliability of resistance detection based on RCV, a correlation analysis was conducted by comparing the mortalities of representative strains obtained by RCV with the diagnostic doses and those by spray with recommend doses. The average correlation coefficient was 0.762 (p < 0.001) (Table 4). However, if excluding cyflumetofen, a relatively slow-acting metabolic inhibitor, all tested acaricides revealed high degrees of correlation (*ρ* > 0.845) between the mortalities determined by RCV and spray methods, suggesting the reliability of RCV method in resistance detection (Table 4 and S4 Table).

**Table 4:** Correlation coefficients between the mortalities obtained by RCV with diagnostic doses and those obtained by spray with recommended doses of six representative acaricides.

Acaricides	Correlation coefficient (*ρ*)	P-value
Monocrotophos	0.893	0.016
Bifenthrin/fenpropathrin*	0.924	0.008
Abamectin	0.898	0.015
Tebufenpyrad	0.845	0.034
Cyflumetofen	0.775	0.070
Bifenazate	1.000	< 0.001
Average	0.762	< 0.001

*The mortalities by RCV with bifenthrin were compared with those by spray with fenpropathrin, another pyrethroid acaricide.

### Establishment of QS regressions for the prediction of allele frequencies

Several equations for the allele frequency predictions have been previously established for the mutations in *Tuace* (G228S and F439W), *Tuvssc* (L1022V) and *TuGluCl1* (G323D) [37]. Along with these, additive QS equations were newly established for the frequency prediction of six point mutations in *Tuvssc* (A1376D and F1704I), *TuGluCl3* (G326E), *TuCHS* (I1017F) and *TuCytB* (G126S and P262T). Although five mutations (G126S, I136T, S141F, D161G and P262T) in *TuCytB* have been reported to be associated with bifenazate resistance, the QS protocol was established for only G126S and P262T mutations as the representative markers for bifenazate resistance because the other three mutations (I136T, S141F, and D161G) have not been identified in the mite populations tested in this study. High correlation coefficients (*r* ^*2*^ = 0.982 ~ 0.999) were obtained in all regression equations generated by plotting the resistant nucleotide signal ratios of each mutation and the corresponding resistance allele frequencies (S5 Table). The lower and higher detection limits ranged from 4.5~21.9% (12.8±5.7%) and 89.1~97.5% (91.8±3.1%) at the 95% confidence level, respectively.

### Determination of resistance allele frequencies in mite populations by QS

QS was employed to determine the frequencies of resistance alleles corresponding to the 12 point mutations that have been reported to date (Fig 2B). The frequencies of all mutations examined were close to 0% in the susceptible reference strains (UD and PyriF). The frequencies of the G228S and F439W mutations in *Tuace*, associated with organophosphate resistance [19], were generally high in most populations including the AD strain. In addition, the G228A and F439Y mutations, other alleles of the G228S and F439W mutations, respectively, were also found in some populations. In the AbaR and 13CB_JC_R1 populations, the G228A mutation was present in higher frequencies than the G228S mutation. The F439Y mutation was only found in the 13JB_GJ_G1 population, which has also been reported in several resistant populations in the Netherlands [50].

Among the three point mutations (L1022V, A1376D and F1704I) that have been reported to be associated with pyrethroid resistance [20, 21]; the L1022V and A1376D mutations were found in similar frequencies in several populations including the FenR strain, suggesting a close linkage between these two loci. In contrast, the F1704I mutation allele was observed in high frequencies only in a few field populations (13GG_GY_G1, 13GG_SW_G1 and 13JB_GJ_G1), which indicates that it has not widespread yet in Korea. Four red-type mites showed 100% frequency of the A1376D mutation. The number of histidine residues in the intracellular loop of the voltage-sensitive sodium channel, which was different between the susceptible and resistant strains [20], varied among different mite strains, suggesting the likelihood of a simple polymorphism without association with pyrethroid resistance in *T*. *urticae* (S6 Table).

Both the G323D and G326E point mutations associated with abamectin resistance, which have been found in *TuGluCl1* and *TuGluCl3*, respectively [22, 23], were found together and saturated (100%) in all red-type mite populations (Fig 2B and S6 Table). Interestingly, one population of green-type mites, 13_GG_GY_G1, was determined to possess only the G326E mutation in *TuGluCl3* without the G323D mutation in *TuGluCl1*, suggesting that these two resistance mutations have been independently selected.

The I1017F mutation in *TuChS1*, which is associated with etoxazole resistance [24] and cross-resistance to several mite growth inhibitors such as hexythiazox and clofentezine [51], was found to be saturated in all red-type mites examined. Three field-collected green-type mite populations (13GG_GY_G1, 13GG_SW_G1 and 23JB_GJ_G1) also possessed allele frequencies of over 80% (Fig 2B and S6 Table). Laboratory strains of green-type mites and 13JB_GJ_G1 did not possess the I1017F mutation.

Out of the two mutations (G126S and P262T) on mitochondrial *TuCytB* reported to be involved in bifenazate resistance, the G126S mutation was found in 13GG_GY_G1, AbaR, and 13CB_JC_R1, whereas the P262T mutation was found in 13GG_SW_G1 and 13GG_GY_R1 (Fig 2B and S6 Table).

### Correlation analysis between RCV immortality and QS resistant allele frequency

Correlation analyses were conducted to investigate the relationships between the actual resistance levels determined by RCV and the resistance allele frequencies obtained by QS. Allele frequencies of the G228S, G228A and F439Y mutations on *Tuace* revealed low correlations (*ρ* = -0.620 ~ 0.475) with the mortality rates of the organophosphate and carbamate acaricides (Table 5). However, the F439W mutation frequencies revealed an increased correlation (*ρ* = 0.539) with the mortality rate by omethoate. Frequencies of the L1022V and A1376D mutations on *Tuvssc* were highly correlated with the mortality rate by bifenthrin [*ρ* = 0.859 (p < 0.01) and *ρ* = 0.671 (p < 0.05), respectively] (Table 5), and tightly linked (*ρ* = 0.930, p < 0.01) (S1 Fig). However, the *Tuvssc* F1704I mutation frequencies revealed a low correlation with L1022V and A1376D (*ρ* = -0.4 and -0.24, respectively) (S1 Fig). The G323D and G326E mutation frequencies were highly correlated with the mortality rate by abamectin (*ρ* = 0.659 and 0.768, respectively) (Table 5). Frequencies of both mutations also revealed a high correlation to each other (*ρ* = 0.953, p < 0.01) (S1 Fig). Frequencies of the *TuChS1* I1017F revealed the highest correlation (*ρ* = 0.970, p < 0.01) with the mortality rate by etoxazole (Table 5). Frequencies of the *TuCytB* G126S and P262T mutations revealed low correlations with the mortality rate by bifenazate (*ρ* = 0.583 and 0.463, respectively). Nevertheless, all strains (13GG_SW_G1 and 13GG_GY_R1) that have the P262T mutation are highly resistant to bifenazate, suggesting that it is a main causative mutation for bifenazate resistance. In case of a strain with neither the G126S nor P262T mutation (13GG_PJ_R1) but exhibiting some level of bifenazate resistance, other metabolic or toxicokinetic resistance mechanisms are likely to be involved.

**Table 5:** Correlation coefficient between allele frequency of mutation and mortality determined by RCV from 12 strains.

Genes	Mutation	Acaricides (Correlation coefficient, *ρ)*						
		Fenothiocarb	Monocrotophos	Omethoate	Bifenthrin	Abamectin	Etoxazole	Bifenazate
Tuace	G228S	-0.620* ^a^	-0.014	-0.057	-0.091	-0.188	-0.075	-0.227
	G228A	0.475	0.413	0.279	0.338	0.434	0.433	0.455
	F439W	0.014	0.539	0.444	0.230	0.506	0.590	0.367
	F439Y	-0.258	0.033	-0.250	-0.014	-0.403	0.382	-0.300
Tuvssc	L1022V	0.759** ^b^	0.436	0.590*	0.859**	0.755**	0.518*	0.772**
	A1376D	0.741**	0.355	0.547	0.671*	0.699*	0.307	0.653*
	F1704I	-0.315	0.159	-0.105	0.237	-0.101	0.463	0.026
TuGluCl1	G326E	0.880**	0.694*	0.824**	0.663*	0.768**	0.782**	0.974**
TuGluCl3	G323D	0.909**	0.622*	0.802**	0.551	0.659*	0.751**	0.848**
TuCHS	I1017F	0.693*	0.727**	0.668*	0.689*	0.569	0.970**	0.835**
TuCytb	G126S	0.381	0.446	0.333	0.454	0.521	0.371	0.583*
	P262T	0.221	0.201	0.506	0.264	0.428	0.536	0.463

^a^ * *P* < 0.05

^b^ ** *P* < 0.01

Interestingly, the frequencies of *TuGluCl* mutation showed high correlations (*ρ* >~0.7, P<0.01) not only with abamectin but with other acaricides acting on completely different target, such as bifenazate, fenothiocarb and etoxazole (Table 5).

## Discussion

Through biochemical and molecular biological approaches to understand pesticide resistance, many types of resistance-evoking factors, mainly governed by target site mutations, have been elucidated [15, 52]. The resistance-associated mutations have been employed as genetic markers to determine the resistance levels in pest populations in conjunction with high-throughput screening strategies for massive samples with decreasing cost that can replace the traditional phenotypic resistance monitoring [53–57]. However, it is important to evaluate the accuracy and representativeness of resistance genetic markers in determining resistance levels in field pest populations showing multiple resistance traits.

To this end, we evaluated the performance of RCV and QS methods as complimentary tools for the detection of acaricide resistance in several field populations of *T*. *urticae*. The reliability of RCV as a resistance monitoring tool was confirmed by comparing the RCV toxicity data with the toxicity data obtained by spray bioassay. In addition, we also assessed the correlation between the RCV vs. spray data and RCV vs. QS data to determine whether allele frequency data generated from QS could be employed as an alternative index to quantify resistance levels. Finally, we examined whether and how different resistance profiles are related each other.

Diagnostic doses for a more inclusive set of fast-acting acaricides were established and employed to determine the resistance levels in several populations of *T*. *urticae* based on RCV. For acaricides with low contact toxicity or slow-acting mite growth regulators, such as etoxazole, fenbutatin oxide, flufenoxuron, spiromesifen (Table 3), RCV was impractical for establishing diagnostic doses and the traditional leaf dipping method was used. When treated with the diagnostic doses using RCV, all reference acaricide-susceptible and -resistant strains showed expected mortality responses, demonstrating the discrimination capacity of the diagnostic doses.

Many field-collected mites showed high levels of resistance to most tested acaricides with RCV, indicating the widespread nature of multiple resistance in these populations and the necessity for immediate action by resistance management. In general, the red-type mites collected from the greenhouse rose cultivation areas exhibited relatively higher resistance levels compared with the green-type mites, suggesting their potential genetic differences in resistance development. The red-type mites clustered with Hap_17 (corresponding lineage I to Hinomoto et al., 2001 [58]) and similar red-type mites have been reported in European and Israeli populations [38], suggesting their recent invasion into the greenhouse rose cultivation areas of Korea. Rapid and comprehensive profiling of resistance (or susceptibility) status by RCV would enable a more efficient selection of optimum alternative acaricides for rotation and would facilitate an understanding of the pattern of resistance development in these invasive, difficult-to-control populations of *T*. *urticae*.

In our previous studies, we established the QS protocol for the determination of resistance allele frequencies associated with resistances to OP, pyrethroid and abamectin in *T*. *urticae* [37]. In this study, we established additional QS protocols for the prediction of the recently identified 12 nonsynonymous point mutations putatively associated with acaricide resistance (S5 Table). QS analysis revealed that the susceptible reference strains (UD and PyriF) exhibited 0% frequency in all the resistance alleles examined, whereas the laboratory acaricide-resistant strains showed 100% or near 100% frequencies in the alleles corresponding to respective resistance traits. These results demonstrate the accuracy of QS in predicting resistance allele frequency. As determined by QS, *T*. *urticae* collected from various rose cultivation greenhouses exhibited high frequencies in nearly all resistance alleles examined, indicating the widespread distribution of multiple resistance in these field populations and further suggesting that they have been extensively selected by a wide variety of acaricides.

Recently, a worldwide distribution of target site mutations associated with acaricide resistance in *T*. *urticae* was also surveyed by sequencing the relevant gene fragments of five target genes [59]. This sequencing analysis for determining the presence or absence of point mutations can provide qualitative information on the distribution and occurrence of resistance alleles in mite populations of different geographical regions but not quantitative information on the resistance allele frequency. With this in mind, QS could be employed as a cost-effective genotyping tool to predict and quantify resistance allele frequencies in multiple numbers of mite populations in the initial stage of resistance monitoring.

The correlation analyses revealed that frequencies of the resistance alleles on respective target sites of some acaricides, such as bifenthrin, abamectin and etoxazole, are relatively well correlated with the mortality responses to the diagnostic doses of those acaricides (*ρ* = 0.655~0.785, 0.723~0.816 and 0.968, respectively). In these cases, the resistance allele frequency can represent the actual resistance level and can be used as an alternative index for resistance monitoring. In the case of the other acaricides examined, however, target site resistance allele frequencies exhibited only low correlations with mortality responses, limiting the use of allele frequency as a direct indicator for estimating actual resistance levels. This low level of correlation is likely, at least in part, due to the fact that the frequencies of the target site resistance alleles are almost saturated in most of the test populations, except for the susceptible reference strains. It is also possible that the other target site resistance factors play more determining roles in resistance. In the case of the *Tuace* mutations, *Tuace* gene amplification functions as an additive factor to resistance and complicates the interpretation of phenotypic resistance levels contributed by the target site mutations [9, 18]. Since metabolic resistance mechanisms (i.e., esterase and cytochrome P450) have been well documented for bifenthrin and ababmectin resistance [60, 61], the presence of metabolic resistance factors in some mite populations likely causes the reduction of correlation. Nevertheless, the resistance allele frequency data alone allowed for the evaluation of the genetic resistance potential and background of test mite populations. In addition, the routine assessment of resistance allele frequencies could provide fundamental information for understanding the temporal and spatial dynamics of resistance alleles. The information on the *T*. *urticae* genome would greatly facilitate the identification of a wide array of novel resistance markers on a genome scale [13], which, in turn, would greatly expand the applicability of QS-based molecular resistance monitoring.

Interestingly, when different resistance traits in the mites collected from rose-cultivating green houses were compared on the bases of resistance allele frequencies on various acaricide target sites, resistance levels to certain acaricides were highly correlated with the mutation allele frequencies that are associated with resistance to other groups of acaricides with completely different modes of action (i.e., fenothiocarb, abamectin and bifenazte vs. *Tuvssc* L1022V mutation; fenothiocarb, omethoate, etoxazole and bifenazate vs *TuGluCl* mutations; monocrotophos and bifenazate vs. *TuCHS* I1017F mutation; P<0.01) (Table 5). These non-specific correlations do not necessarily indicate the presence of a cross-resistance mechanism between such different groups of acaricides, but rather reflect the simultaneous development of resistance due to a common use history of the acaricides clustered together. Considering that various acaricides are used together with high selection pressures for mite control and the introgression of susceptible alleles is extremely limited in a closed greenhouse environment, the rapid and concurrent accumulation of multiple resistance alleles in the acaricide-selected mites would be natural in the greenhouse environment.

## Conclusions

Rapid resistance monitoring is the utmost prerequisite for the proper management of acaricide-resistant populations of *T*. *urticae*. RCV can be employed as a rapid tool for the on-site monitoring of resistance levels to fast-acting acaricides. However, to maximize the applicability of RCV, the determination of diagnostic doses for a wider array of acaricides is necessary. As a tool for the genotyping of resistance alleles on a population basis, QS protocols were established, and their potential as a molecular resistance monitoring tool was evaluated. The resistance allele frequencies estimated by QS showed high levels of correlation with actual resistance levels for only a few types of acaricides, limiting the use of allele frequency as a direct indicator to estimate actual resistance level to all types of acaricides. Nevertheless, the resistance allele frequency data can provide crucial information for assessing the genetic resistance potential and background and for understanding the temporal and special dynamics of resistance alleles. With this in mind, a genome-wide search for a more inclusive repertoire of molecular resistance markers that can be used for QS is required for the wider application of QS in resistance detection. In conjunction with the RCV bioassay, the complementary use of QS will greatly contribute to the management of *T*. *urticae* acaricide resistance.

## Supporting Information

S1 Fig Spearman correlation analysis to determine the correlation between allele frequencies in *T*. *urticae* strains. The circle size is proportional to the value of Spearman's rank correlation coefficient. The ‘X’ denotes a p-value higher than 0.05 in the confidence interval.
(DOCX)

S1 Table GenBank ID used for the construction of phylogenetic tree based on mtCOI. (DOCX)

S2 Table Oligonucleotide primers used in this study. Bold characterized primers were used for sequencing.
(DOCX)

S3 Table Mortalities of 12 strains of *T*. *urticae* to 12 acaricides when treated with the diagnostic doses via RCV. (DOCX)

S4 Table Comparison of mortalities between RCV and spray methods. (DOCX)

S5 Table Regression and prediction equations for the estimation of resistance allele frequencies. (DOCX)

S6 Table Allele frequencies associated with acaricide resistance in *T*. *urticae* strains determined by QS. (DOCX)
