# Supplementary material for: Phenotypic- and Genotypic-Resistance Detection for Adaptive Resistance Management in Tetranychus urticae Koch
Source: PLoS One. 2015 Nov 6;10(11):e0139934. doi: 10.1371/journal.pone.0139934 (PMC4636269; doi:10.1371/journal.pone.0139934)

**S1 Fig. Spearman correlation analysis to determine the correlation between allele frequencies in mite strains.** The circle size is proportional to the value of Spearman's rank correlation coefficient. The ‘X’ denotes a p-value higher than 0.05 in the confidence interval.
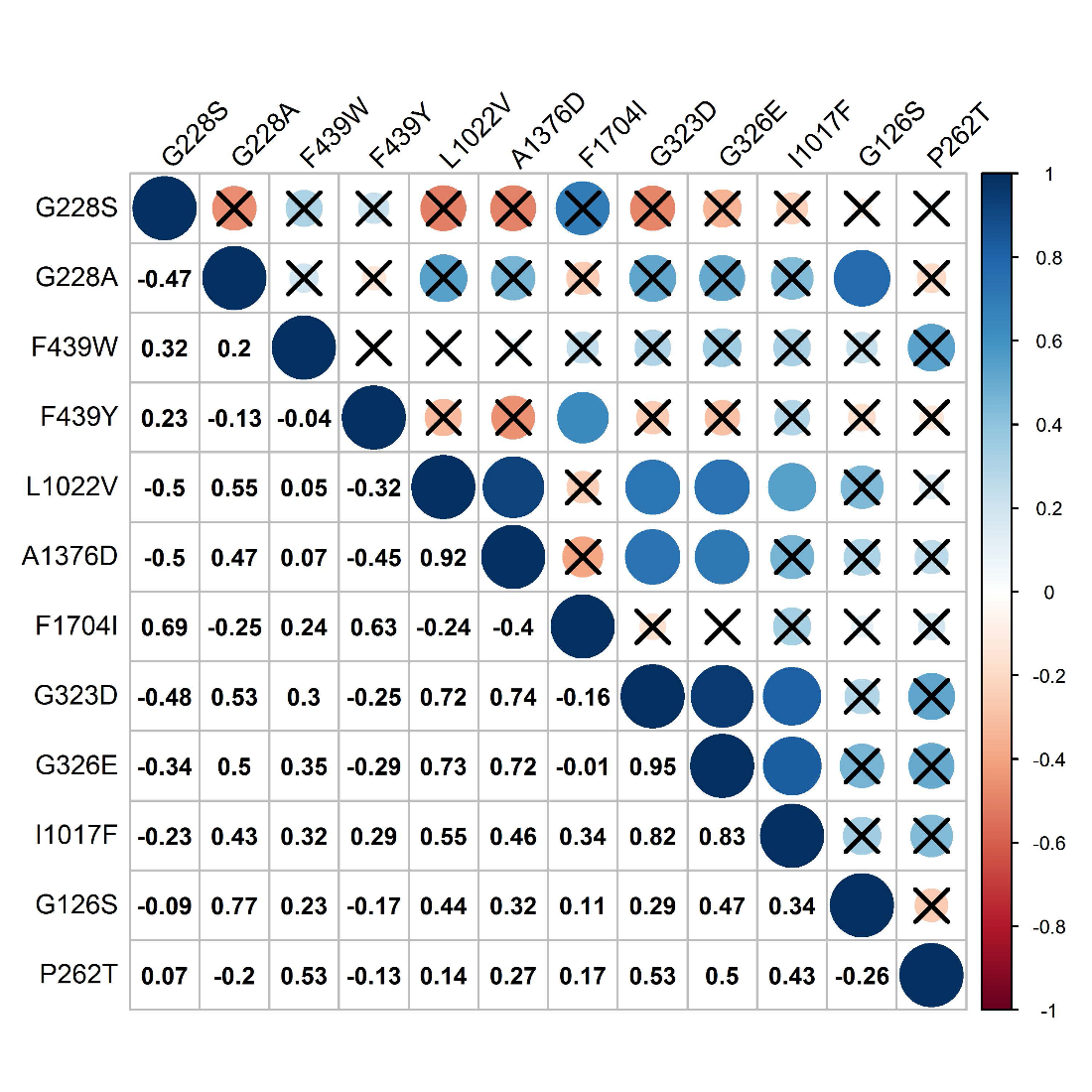

Supplement: S1 Fig — The circle size is proportional to the value of Spearman's rank correlation coefficient. The ‘X’ denotes a p-value higher than 0.05 in the confidence interval. (DOCX) [file pone.0139934.s001.docx]
