# Supplementary material for: Phenotypic- and Genotypic-Resistance Detection for Adaptive Resistance Management in Tetranychus urticae Koch
Source: PLoS One. 2015 Nov 6;10(11):e0139934. doi: 10.1371/journal.pone.0139934 (PMC4636269; doi:10.1371/journal.pone.0139934)
Supplement: S1 Table — (DOCX) [file pone.0139934.s002.docx]

**S1 Table. GenBank ID used for the construction of phylogenetic tree based on mitochondrial cytochrome c oxidase subunit I (COI).**

| Species | GenBank ID and strain name | Haplotype | Reference |
| --- | --- | --- | --- |
| *Oligonichus cofeae* | AB683671.1_OC0025 |  | Matsuda et al., 2013 |
| *Oligonichus gotohi* Ehara | AB683668.1_OG0076 |  | Matsuda et al., 2013 |
| *Tetranychus evansi* | AB736039.1_Tev0210, AB736040.1_Tev0211 |  | Matsuda et al., 2013 |
| *Tetranychus kanzawai* | AB736043.1_Tka0158, AB736044.1_Tka0159, AB736045.1_Tka0166, AB736046.1_Tka0010, AB736047.1_Tka0247, AB736048.1_Tka0249, B736049.1_Tka0250, AB736050.1_Tka0385 |  | Matsuda et al., 2013 |
| *Tetranychus ludeni* | AB736051.1_Tlu0189, AB736052.1_Tlu0017, AB736053.1_Tlu0275 |  | Matsuda et al., 2013 |
| *T. misumaiensis* Ehara & Gotoh | AB736054.1_Tmi0218 |  | Matsuda et al., 2013 |
| *T. neocaledonicus* | AB736055.1_Tne0192, AB736056.1_Tne0003, AB736057.1_Tne0008 |  | Matsuda et al., 2013 |
| *T. okinawanus* Ehara | AB736058.1_Tok0208, AB736059.1_Tok0373 |  | Matsuda et al., 2013 |
| *T. phaselus* Ehara | AB736066.1_Tph0191, AB736067.1_Tph0338 |  | Matsuda et al., 2013 |
| *T. piercei* McGregor | AB736068.1_Tpi0014, AB736069.1_Tpi0063, AB736070.1_Tpi0397 |  | Matsuda et al., 2013 |
| *T. pueraricola* Ehara & Gotoh | AB736071.1_Tpu0203, AB736072.1_Tpu0205, AB736073.1_Tpu0204 |  | Matsuda et al., 2013 |
| *T. truncatus* Ehara | AB736074.1_Ttr0196, AB736075.1_Ttr0195 |  | Matsuda et al., 2013 |
| *T. urticae* Koch | AB736076.1_TuG0181, AB736077.1_TuG0185, AB736078.1_TuG0188, AB116573.1_HAP_1_(G) | HAP_1 | Mendoca et al., 2011 & Matsuda et al., 2013 |
|  | AB736079.1_TuR0171, AB116574.1_HAP_2_(R) | HAP_2 | Mendoca et al., 2011 & Matsuda et al., 2013 |
|  | AB736080.1_TuR0173 | ND | Matsuda et al., 2013 |
|  | AB736081.1_TuR0174 | ND | Matsuda et al., 2013 |
|  | AJ414582.1_HAP_3_(G) | HAP_3 | Mendoca et al., 2011 |
|  | AJ316597.1_HAP_6_(Un) | HAP_6 | Mendoca et al., 2011 |
|  | AJ316598.1_HAP_7_(G) | HAP_7 | Mendoca et al., 2011 |
|  | AJ316599.1_HAP_8_(R) | HAP_8 | Mendoca et al., 2011 |
|  | AJ316605.1_HAP_9_(Un) | HAP_9 | Mendoca et al., 2011 |
|  | AJ316606.1_HAP_10_(Un) | HAP_10 | Mendoca et al., 2011 |
|  | AJ316600.1_HAP_11_(Un) | HAP_11 | Mendoca et al., 2011 |
|  | HM565892.1_HAP_12_(R) | HAP_12 | Mendoca et al., 2011 |
|  | HM565893.1_HAP_14_(R) | HAP_14 | Mendoca et al., 2011 |
|  | HM565898.1_HAP_15_(R) | HAP_15 | Mendoca et al., 2011 |
|  | FJ594469.1_HAP_17_(R) | HAP_17 | Mendoca et al., 2011 |
|  | HM565896.1_HAP_18_(R) | HAP_18 | Mendoca et al., 2011 |
|  | KF977427 _UD | HAP_1 | Kwon et al., 2011 |
|  | KF977426 _PyriF | HAP_1 | Kwon et al., 2011 |
|  | KF977423 _AD | HAP_1 | Kwon et al., 2011 |
|  | KF977424 _FenR | HAP_1 | Kwon et al., 2011 |
|  | KF977425 _PTF | HAP_1 | Kwon et al., 2011 |
|  | KF977416 _13GG_GY_G1 | HAP_1 | In this study |
|  | KF977418 _13GG_SW_G1 | HAP_1 | In this study |
|  | KF977419 _13JB_GJ_G1 | HAP_1 | In this study |
|  | KF977428 _AbaR | ND | Kwon et al., 2011 |
|  | KF977415 _13GG_GY_R1 | ND | In this study |
|  | KF977417 _13GG_PJ_R1 | ND | In this study |
|  | KF977413 _13CB_JC_R1 | ND | In this study |

ND, not determined; (G), Green form; (R), Red form; (Un), not reported form
