# Supplementary material for: Phenotypic- and Genotypic-Resistance Detection for Adaptive Resistance Management in Tetranychus urticae Koch
Source: PLoS One. 2015 Nov 6;10(11):e0139934. doi: 10.1371/journal.pone.0139934 (PMC4636269; doi:10.1371/journal.pone.0139934)
Supplement: S2 Table — Bold characterized primers were used for sequencing. (DOCX) [file pone.0139934.s003.docx]

**S2 Table. Oligonucleotide primers used in this study. Bold characterized primers were used for sequencing.**

| Target gene | Mutation | Oligonucleotide | Oligonucleotide sequence | Remarks |
| --- | --- | --- | --- | --- |
| *Tuace* | G228S  F439W | 5'TSSMace1geno | TATGGGTGCCACCGAATGGAA | Kwon et al., 2010 |
|  |  | 3'TSSMace1geno | CTAAAGGACTCAGATGGGGAT |  |
|  |  | **5'TSSMFWgenoF** | AAAGCCTATCAACGGAGCCTT |  |
|  |  | **3'TSSMGSgenoR** | TGTTTGCCGGATTACCACCAA |  |
| *Tuvssc* | L1022V | 5’TSSMIIS4GSP | TCCGAATGCAGAGATTCCTCGTT | Kwon et al., 2013 |
|  |  | 3’TSSMIIS6IIIS1 | ACCACTTGATGCACCACCTCTA |  |
|  |  | **3’TSSMscgQS(N)** | TGCCGTGTCTCGAATCTGATT |  |
|  | A1376D | 131021_1F | GGCAACATTCAAAGGTTGGAC |  |
|  |  | 131021_1R | ACATTCCAAGGTTCCCGGAAA |  |
|  |  | **131021_1R_mutdet** | TGAGCATGCATTCAGCAGTG |  |
|  | F1704I | 130611_2F | TCACCCTTTTTCAGTGGACA |  |
|  |  | 130611_2R | CCTGGAACCACCTCCTTTCT |  |
|  |  | **130611_2F_mutdet** | GATTGCCTATTTTGCCCTGA |  |
| *TuGluCl3* | G323D | 5'GluGC3'RACE(N) | GGGGAATACAGCTGTCTCAAA | Kwon et al., 2013 |
|  |  | 3'TSSMGluQS | TGAATCCTTGGCGGTGTCAAA |  |
|  |  | **3’TSSMGluQS(N)** | ACGCCGATGTTTATCAGCTTT |  |
| *TuGluCl1* | G326E | 131106_1F | GGCAACTCAAATATCCGGTAT |  |
|  |  | 131106_1R | TGAGGCTCTTGATGAATGGAA |  |
|  |  | **131106_1R_det** | TCTGGGTTTCAAGTCTACACG |  |
| *TuCHS* | I1017F | 130611_3F | CAAGCGAACTGTGGCAGTAA |  |
|  |  | 130611_3R | CTTTTCGTCGTTTGGTTTGG |  |
|  |  | **130611_3F_mutdet** | CGGAGCTATGGTTGCTGTCTT |  |
| *TuCytB* | G126S/P262T | Cytbdia2F | TTAAGAACTCCTAAAACTTTTCGTTC | Van Nieuwenhuyse et al., 2008 |
|  |  | 130611_4R_mutdet | GAAACAAAAATTATTATTCCCCCAAC |  |
|  |  | **CytbWTF** | CGGAATAATTTTACAAATAACTCATGC |  |
|  |  | **PEWYF1** | AAAGGCTCATCTAACCAAATAGG |  |
