# Supplementary material for: Phenotypic- and Genotypic-Resistance Detection for Adaptive Resistance Management in Tetranychus urticae Koch
Source: PLoS One. 2015 Nov 6;10(11):e0139934. doi: 10.1371/journal.pone.0139934 (PMC4636269; doi:10.1371/journal.pone.0139934)
Supplement: S3 Table — (DOCX) [file pone.0139934.s004.docx]

**S3 Table. Mortality of 12 populations to 12 acaricides by using residual contact vial bioassay which was coated with diagnostic doses.**

| Acaricides | Mortality of field strains | | | | | | | | | | | |
| --- | --- | --- | --- | --- | --- | --- | --- | --- | --- | --- | --- | --- |
|  | UD | PyriF | AD | FenR | PTF | 13GG_  GY_G1 | 13GG_  SW_G1 | 13JB_  GJ_G1 | AbaR | 13GG_  GY_R1 | 13GG_  PJ_R1 | 13CB_  JC_R1 |
| Fenothiocarb | 100±0 d | 100±0 d | 91.9±7 cd | 100±0 d | 100±0 d | 91.1±3.8 cd | 75.6±3.8 c | 100±0 d | 49.2±10 b | 50±3.3 b | 24.8±19 a | 34.3±13.9 ab |
| Monocrotophos | 100±0d c | 100±0d c | 5.9±5.9 a | 100±0 c | 88.4±15 c | 28.4±6.9 ab | 30.4±10 ab | 43±10.7 b | 4.2±7.2 a | 30.5±23.3 ab | 11.1±3.8 a | 22.8±9.1 ab |
| Omethoate | 100±0 e | 100±0 e | 27.7±17.7 abc | 100±0 e | 96.5±6.1 de | 56.3±13.9 cd | 0±0 a | 86.7±23.1 de | 41.8±14.7 bc | 26±17.3 abc | 27.9±25.3 abc | 3.5±3.1 ab |
| Endosulfan | 100±0 d | 100±0 d | 100±0 d | 100±0 d | 100±0 d | 73.3±0 bc | 62.4±2.4 b | 71.1±5.6 bc | 89.2±10.1 cd | 56.5±6.3 b | 32.1±16.3 a | 29.9±5.2 a |
| Bifenthrin | 100±0 e | 100±0 e | 100±0 e | 6.3±6.3 a | 97.6±4.1 e | 17.8±7.7 ab | 15.4±11.8 ab | 52.5±10.9 d | 15.5±9 ab | 43.2±10 cd | 28.1±8.8 abc | 33±5.9 bcd |
| Abamectin | 100±0 d | 100±0 d | 100±0 d | 47.1±5.1 c | 26.5±6.7 abc | 15.6±10.2 ab | 15.5±5.2 ab | 100±0 d | 0±0 a | 7.5±0.7 a | 43.3±29.5 bc | 25.2±9.4 abc |
| Etoxazole | 100±0 d | 100±0 d | 100±0 d | 100±0 d | 100±0 d | 49.3±16.4 c | 0±0a | 6.7±2.6 ab | 0±0 a | 20.0±12.4 a | ND | ND |
| Chlofenapyr | 100±0 d | 100±0 d | 100±0 d | 95.6±3.8 d | 91.9±7 d | 87.7±6 cd | 12.3±0.4 a | 100±0 d | 74.5±9 bc | 73.4±6.1 b | 66.8±5.7 b | 15.6±4.5 a |
| Tebufenpyrad | 100±0 c | 100±0 c | 98±3.4 c | 100±0 c | 100±0 c | 62.9±2.5 ab | 60.4±24.6 ab | 100±0 c | 57±11.5 a | 86±1.2 bc | 62.2±15.4 ab | 49±12.2 a |
| Cyenopyrafen | 100±0 d | 100±0 d | 100±0 d | 100±0 d | 100±0 d | 74.7±12.1 cd | 11.1±7.7 a | 100±0 d | 66.6±4 bc | 68.8±4.5 bcd | 20±11.5 a | 39.2±31.2 ab |
| Cyflumetofen | 100±0 d | 100±0 d | 98.0±3.4 d | 100±0 d | 100±0 d | 100±0 d | 14.6±15.7 a | 100±0 d | 91.3±3 d | 61.6±18.7 c | 41.8±16.5 bc | 23.9±5.5 ab |
| Bifenazate | 100±0 b | 100±0 b | 100±0 b | 100±0 b | 100±0 b | 11.6±4.6 a | 2.1±3.6 a | 100±0 b | 0±0 a | 13.7±13.3 a | 15.4±10.1 a | 15.6±9.6 a |
