# Supplementary material for: Phenotypic- and Genotypic-Resistance Detection for Adaptive Resistance Management in Tetranychus urticae Koch
Source: PLoS One. 2015 Nov 6;10(11):e0139934. doi: 10.1371/journal.pone.0139934 (PMC4636269; doi:10.1371/journal.pone.0139934)
Supplement: S4 Table — (DOCX) [file pone.0139934.s005.docx]

| Application method | Strain | Acaricides (Mortality, %) | | | | | |
| --- | --- | --- | --- | --- | --- | --- | --- |
|  |  | Monocrotophos | Bifenthrin | Abamectin | Tebufenpyrad | Cyflumetofen | Bifenazate |
| RCV | UD | 100±0 | 100±0 | 100±0 | 100±0 | 100±0 | 100±0 |
|  | PyriF | 100±0 | 100±0 | 100±0 | 100±0 | 100±0 | 100±0 |
|  | AD | 5.9±5.9 | 100±0 | 100±0 | 98±3.4 | 98.0±3.4 | 100±0 |
|  | FenR | 100±0 | 6.3±6.3 | 47.1±5.1 | 100±0 | 100±0 | 100±0 |
|  | PTF | 88.4±15 | 97.6±4.1 | 26.5±6.7 | 100±0 | 100±0 | 100±0 |
|  | AbaR | 4.2±7.2 | 15.5±9 | 0±0 | 57±11.5 | 91.3±3 | 0±0 |
| Spray | UD | 100±0 | 100±0^*^ | 100±0 | 100±0 | 100±0 | 100±0 |
|  | PyriF | 91.7±14.4 | 98.3±2.9^*^ | 100±0 | 95±8.7 | 100±0 | 100±0 |
|  | AD | 0.2±0.4 | 81±7.5^*^ | 100±0 | 79.1±6.5 | 100±0 | 100±0 |
|  | FenR | 100±0 | 1.8±3^*^ | 100±0 | 92.6±3.8 | 100±0 | 100±0 |
|  | PTF | 41.7±41.6 | 75.1±13^*^ | 93.5±6.7 | 94.2±0.7 | 100±0 | 100±0 |
|  | AbaR | 3.7±6.4 | 1.8±3^*^ | 3.9±6.8 | 1.7±2.9 | 71.7±30.1 | 18.1±6.6 |

**S4 Table. The comparison of mortality between RCV and spray methods.**

* Asterisk represent the data determined by fenpropathrin acaricides including same class with bifenthrin.
