# Supplementary material for: Phenotypic- and Genotypic-Resistance Detection for Adaptive Resistance Management in Tetranychus urticae Koch
Source: PLoS One. 2015 Nov 6;10(11):e0139934. doi: 10.1371/journal.pone.0139934 (PMC4636269; doi:10.1371/journal.pone.0139934)
Supplement: S5 Table — (DOCX) [file pone.0139934.s006.docx]

**S5 Table. Regression and prediction equations for the estimation of resistance allele frequencies.**

| Target site | Mutation | Sequencing direction | Regression  equation ^a^ | Regression coefficient (*r^2^*) | Prediction equation (at 95% CL) | |
| --- | --- | --- | --- | --- | --- | --- |
|  |  |  |  |  | Lower | Upper |
| *Tuace1* | G228S^b^ | Antisense | *y* = -4.0+104.5*x* | 0.993 | *y* = -14.4+104.6*x* | *y* = 6.4+104.4*x* |
| *Tuace1* | F439W^b^ | Sense | *y* = -0.1+101.2*x* | 0.999 | *y* = -3.9+101.2*x* | *y* = 3.7+101.2*x* |
| *Tuvssc* | L1022V^b^ | Antisense | *y* = 3.4+95.8*x* | 0.998 | *y* = -6.3+ 95.7*x* | *y* = 13.1+95.9*x* |
| *Tuvssc* | A1376D | Sense | *y* = 6.2+99.4*x* | 0.982 | *y* = -9.5+98.6*x* | *y* = 21.9+100.3*x* |
| *Tuvssc* | F1704I | Sense | *y* = -0.2+102.3*x* | 0.999 | *y* = -4.8+102.3*x* | *y* = 4.5+102.3*x* |
| *Tuglucl1* | G323D^b^ | Antisense | *y* = 2.5+97.8*x* | 0.994 | *y* = -3.5+97.7*x* | *y* = 8.4+97.8*x* |
| *Tuglucl3* | G326E | Antisense | *y* = -1.5+104.7*x* | 0.988 | *y* = -14.8+104.6*x* | *y* = 11.8+104.8*x* |
| *TuCHS* | I1017F | Sense | *y* = -2.9+101.3*x* | 0.991 | *y* = -8.5+101.0*x* | *y* = 14.2+101.7*x* |
| TuCytb | G126S | Sense | *y* = 3.1+99.9*x* | 0.992 | *y* = -7.7+99.6*x* | *y* = 13.9+100.2*x* |
| TuCytb | P262T | Sense | *y* = 2.0+5.3*x*+91.6*x^2^* | 0.997 | *y* = -6.5+6.6*x*+90.0*x^2^* | *y* = 10.34+3.9*x*+92.3*x^2^* |

^a^ In the equation, *y* and *x* represent the % resistance allele frequency and resistant nucleotide signal ratio [resistant nucleotide signal/(resistant nucleotide signal + susceptible nucleotide signal)], respectively.

^b^ The equations have been published elsewhere (Kwon et al. 2010b and 2014).
