# Supplementary material for: Phenotypic- and Genotypic-Resistance Detection for Adaptive Resistance Management in Tetranychus urticae Koch
Source: PLoS One. 2015 Nov 6;10(11):e0139934. doi: 10.1371/journal.pone.0139934 (PMC4636269; doi:10.1371/journal.pone.0139934)
Supplement: S6 Table — (DOCX) [file pone.0139934.s007.docx]

**S6 Table. The allele frequencies associated with acaricides resistance from each strains determined by quantitative sequencing.**

| Target site | Mutations | Allele frequencies of strains) | | | | | | | | | | | |
| --- | --- | --- | --- | --- | --- | --- | --- | --- | --- | --- | --- | --- | --- |
|  |  | **UD** | **PyriF** | **AD** | **FenR** | **PTF** | **13GG_GY_G1** | **13GG_SW_G1** | **13JB_GJ_G1** | **AbaR** | **13GG_GY_R1** | **13GG_PJ_R1** | **13CB_JC_R1** |
| TuAChE | **G228S/A** | -4.0  [-14.4 ~ 6.4]^a^ | 49.8  [39.5 ~ 60.2] | 80.8  [70.5 ~ 91.1] | 36.6  [26.3 ~ 47] | 46.1  [35.8 ~ 56.5] | 86.6  [76.3 ~ 96.9] | 82.3  [72 ~ 92.6] | 52.2  [41.8 ~ 62.5] | -4.0/0.87^b^  [-14.4 ~ 6.4] | -4.0  [-14.4 ~ 6.4] | -4.0  [-14.4 ~ 6.4] | -4.0/0.386  [-14.4 ~ 6.4] |
|  | **F439W/Y** | -3.9  [-3.9 ~ 3.7] | -3.9  [-3.9 ~ 3.7] | 97.3  [97.3 ~ 104.9] | 26.4  [26.4 ~ 34] | 75.8  [75.8 ~ 83.4] | 87  [87 ~ 94.6] | 97.3  [97.3 ~ 104.9] | 57.6/0.286  [57.6 ~ 65.2] | 97.3  [97.3 ~ 104.9] | 97.3  [97.3 ~ 104.9] | -3.9  [-3.9 ~ 3.7] | 34.7  [34.7 ~ 42.3] |
| TuVSSC | **L1022V** | 3.4  [-6.3 ~ 13.1] | 3.4  [-6.3 ~ 13.1] | 3.4  [-6.3 ~ 13.1] | 99.2  [89.4 ~ 109] | 3.4  [-6.3 ~ 13.1] | 52.6  [42.9 ~ 62.4] | 82.7  [73.0 ~ 92.5] | 3.4  [-6.3 ~ 13.1] | 99.2  [89.4 ~ 109] | 97.1  [87.3 ~ 106.9] | 99.2  [89.4 ~ 109] | 99.2  [89.4 ~ 109] |
|  | **A1376D** | 6.2  [-9.5 ~ 21.9] | 55.9  [39.8 ~ 72.1] | 39.3  [23.4 ~ 55.3] | 105.6  [89.1 ~ 122.2] | 41.7  [25.7 ~ 57.7] | 47.3  [31.3 ~ 63.4] | 83.2  [66.9 ~ 99.6] | 6.2  [-9.5 ~ 21.9] | 105.6  [89.1 ~ 122.2] | 105.6  [89.1 ~ 122.2] | 105.6  [89.1 ~ 122.2] | 105.6  [89.1 ~ 122.2] |
|  | **His Del^c^** | 6H | 5H | 5H | 4H | 5H | 5H | 4H | 6H | 4H | 4H | 4H | 4H |
|  | **F1704I** | -0.2  [-4.8 ~ 4.5] | -0.2  [-4.8 ~ 4.5] | -0.2  [-4.8 ~ 4.5] | -0.2  [-4.8 ~ 4.5] | -0.2  [-4.8 ~ 4.5] | 82.6  [78 ~ 87.3] | 28.8  [24.2 ~ 33.5] | 102.1  [97.5 ~ 106.8] | -0.2  [-4.8 ~ 4.5] | -0.2  [-4.8 ~ 4.5] | -0.2  [-4.8 ~ 4.5] | -0.2  [-4.8 ~ 4.5] |
| TuGluCl1 | **G323D** | 2.5  [-3.5 ~ 8.4] | 2.5  [-3.5 ~ 8.4] | 2.5  [-3.5 ~ 8.4] | 2.5  [-3.5 ~ 8.4] | 2.5  [-3.5 ~ 8.4] | 2.5  [-3.5 ~ 8.4] | 100.3  [94.2 ~ 106.2] | 2.5  [-3.5 ~ 8.4] | 100.3  [94.2 ~ 106.2] | 100.3  [94.2 ~ 106.2] | 100.3  [94.2 ~ 106.2] | 100.3  [94.2 ~ 106.2] |
| TuGluCl3 | **G326E** | -1.5  [-14.8 ~ 11.8] | -1.5  [-14.8 ~ 11.8] | -1.5  [-14.8 ~ 11.8] | -1.5  [-14.8 ~ 11.8] | -1.5  [-14.8 ~ 11.8] | 61.8  [48.4 ~ 75.2] | 103.2  [89.8 ~ 116.6] | -1.5  [-14.8 ~ 11.8] | 103.2  [89.8 ~ 116.6] | 103.2  [89.8 ~ 116.6] | 103.2  [89.8 ~ 116.6] | 103.2  [89.8 ~ 116.6] |
| TuCHS | **I1017F** | -2.9  [-8.5 ~ 14.2] | -2.9  [-8.5 ~ 14.2] | -2.9  [-8.5 ~ 14.2] | -2.9  [-8.5 ~ 14.2] | -2.9  [-8.5 ~ 14.2] | 87.5  [81.6 ~ 105] | 98.4  [92.5 ~ 115.9] | 98.4  [92.5 ~ 115.9] | 98.4  [92.5 ~ 115.9] | 98.4  [92.5 ~ 115.9] | 98.4  [92.5 ~ 115.9] | 98.4  [92.5 ~ 115.9] |
| CytB | **G126S** | 3.1  [-7.7 ~ 13.9] | 3.1  [-7.7 ~ 13.9] | 3.1  [-7.7 ~ 13.9] | 3.1  [-7.7 ~ 13.9] | 3.1  [-7.7 ~ 13.9] | 103  [91.9 ~ 114.1] | 3.1  [-7.7 ~ 13.9] | 3.1  [-7.7 ~ 13.9] | 103  [91.9 ~ 114.1] | 3.1  [-7.7 ~ 13.9] | 3.1  [-7.7 ~ 13.9] | 103  [91.9 ~ 114.1] |
|  | **P262T** | 2.0  [-6.5 ~ 10.3] | 2.0  [-6.5 ~ 10.3] | 2.0  [-6.5 ~ 10.3] | 2.0  [-6.5 ~ 10.3] | 2.0  [-6.5 ~ 10.3] | 2.0  [-6.5 ~ 10.3] | 98.9  [90.1 ~ 106.5] | 2.0  [-6.5 ~ 10.3] | 2.0  [-6.5 ~ 10.3] | 98.9  [90.1 ~ 106.5] | 2.0  [-6.5 ~ 10.3] | 2.0  [-6.5 ~ 10.3] |

a The number of bracket represent the range of allele frequencies in 95% prediction levels which was deduced from regression curve described in S5 Table.

b The number behind of slash represent the signal ratio of corresponding allele that are located in same amino acid positions.

c His Del represent the number of histidine between intracellular loop between IIS6 and IIIS1 in Tuvssc
